# A nomogram based on systemic inflammation response index and clinical risk factors for prediction of short-term prognosis of very elderly patients with hypertensive intracerebral hemorrhage

**DOI:** 10.3389/fmed.2025.1535443

**Published:** 2025-03-28

**Authors:** Shen Wang, Ruhai Wang, Xianwang Li, Xin Liu, Jianmei Lai, Hongtao Sun, Haicheng Hu

**Affiliations:** ^1^The First School of Clinical Medical, Lanzhou University, Lanzhou, China; ^2^Tianjin Key Laboratory of Neurotrauma Repair, Characteristic Medical Center of People’s Armed Police Forces, Tianjin, China; ^3^Department of Neurosurgery, Fuyang Fifth People’s Hospital, Fuyang, Anhui, China; ^4^Department of Rehabilitation Medicine, Fuyang Fifth People’s Hospital, Fuyang, Anhui, China; ^5^Department of Neurosurgery, Linquan County People’s Hospital, Fuyang, Anhui, China

**Keywords:** very elderly hypertensive intracerebral hemorrhage, poor prognosis, systemic inflammation response index, risk factors, nomogram

## Abstract

**Objective:**

To develop and validate a nomogram based on systemic inflammation response index (SIRI) and clinical risk factors to predict short-term prognosis in very elderly patients with hypertensive intracerebral hemorrhage (HICH).

**Methods:**

A total of 324 very elderly HICH patients from January 2017 to June 2024 were retrospectively enrolled and randomly divided into two cohorts for training (*n* = 227) and validation (*n* = 97) according to the ratio of 7:3. Independent predictors of poor prognosis were analyzed using univariate and multivariate logistic regression analyses. Furthermore, a nomogram prediction model was built. The area under the receiver operating characteristic curves (AUC), calibration plots and decision curve analysis (DCA) were used to evaluate the performance of the nomogram in predicting the prognosis of very elderly HICH.

**Results:**

By univariate and stepwise multivariate logistic regression analyses, GCS score (*p* < 0.001), hematoma expansion (*p* = 0.049), chronic obstructive pulmonary disease (*p* = 0.010), and SIRI (*p* = 0.005) were independent predictors for the prognosis in very elderly patients with HICH. The nomogram showed the highest predictive efficiency in the training cohort (AUC = 0.940, 95% CI: 0.909 to 0.971) and the validation cohort (AUC = 0.884, 95% CI: 0.813 to 0.954). The calibration curve indicated that the nomogram had good calibration. DCA showed that the nomogram had high applicability in clinical practice.

**Conclusion:**

The nomogram incorporated with the SIRI and clinical risk factors has good potential in predicting the short-term prognosis of very elderly HICH.

## Introduction

Hypertensive intracerebral hemorrhage (HICH) is one of the common diseases in neurosurgery, which refers to hemorrhage caused by the rupture of blood vessels in the brain parenchyma, with high rates of mortality and poor functional outcome (1). It accounts for approximately 17.1–55.4% of all strokes (2). Currently, surgical treatment is one of the main treatments for HICH, which can reduce intracranial pressure and thus minimize the extent of damage to brain tissue (3).

ICH incidence increases with age, posing a higher assistance burden in countries with aging population (4–6). However, besides age, a large number of factors need to be considered for risk stratification, including comorbidities, ICH volume and site, and neurological status (5). In recent literature, there have been reports of a correlation between systemic inflammation and poor outcomes in patients with ICH (7, 8). Studies have indicated that systemic inflammation and immune response may exert an indispensable role in brain injury and post-stroke recovery (9). The systemic inflammation triggers secondary injury mechanisms after ICH, which directly contribute to the unfavorable prognosis of patient (10, 11).

The systemic inflammation response index (SIRI) index is a novel inflammatory biomarker, which includes peripheral lymphocytes, neutrophils and monocytes, has been described (12). SIRI index has been shown to be more sensitive for predicting outcome, particularly in cancer patients, than other available methods that use only one or two cell subtypes (13). The predictive value of SIRI index for short-term functional outcome in patients with HICH, however, has not been fully studied. In clinical practice, numerous predictive models have been developed in an attempt to accurately predict the prognosis of HICH (14–16). Wang et al.’s research revealed that a nomogram model consisting of the black hole sign, Glasgow Coma Scale (GCS) score, midline shift, hematoma volume, and Rad-score can accurately predict the short-term prognosis of patients with HICH (15). However, nomograms based on SIRI and clinical risk factors from very elderly HICH patients have not been reported, and their effectiveness in predicting short-term prognosis remains questionable. Therefore, this study attempted to develop and validate a nomogram model based on SIRI and clinical risk factors for predicting poor outcome in very elderly patients with HICH.

## Methods

### Study population

This was a multicenter retrospective study conducted at two hospitals in Anhui, China (Fuyang Fifth People’s Hospital and Linquan County People’s Hospital). Very elderly (≥80 years old) patients with HICH admitted from January 2017 to June 2024 were enrolled in the study. The patients included in the study were divided into two groups using random allocation: a training set consisting of 227 patients and a validation set consisting of 97 patients, with a ratio of 7:3. This study was approved by the ethics committee of Fuyang Fifth People’s Hospital (Ethics approval number: NO.2023052). The inclusion criteria for this study were as follows: age ≥ 80 years, with a history of hypertension or a diagnosis of hypertension during this hospitalization. Hypertension was defined by systolic blood pressure ≥ 140 mmHg, or diastolic blood pressure ≥ 90 mmHg (17); clinically diagnosed with ICH by computed tomography (CT); and admitted to the hospital within 24 h of symptom onset. The exclusion criteria were as follows: ICH caused by head trauma, cerebral aneurysm, vascular malformation, and brain tumor; long-term use of antiplatelet or anticoagulant drugs; used immunosuppressants; had active infection within the 2 weeks before admission; missing imaging data; previous history of ICH or other neurological diseases such as ischemic stroke; other systematic diseases such as renal dysfunction, hepatic dysfunction, cancer, and hematological disorders; incomplete baseline clinical data; and patients who refused to follow-up clinical assessment after being discharged from hospital.

### Data collection

The following data were collected from the patients: gender, age, time from symptom onset to baseline CT scan, history of diabetes and/or coronary heart disease, GCS score at admission, body temperature, pulse, blood pressure at admission (systolic and diastolic blood pressure), location of hematoma, hematoma volume, perihematomal edema volume, modified Graeb Score (mGS), hematoma expansion (HE), chronic obstructive pulmonary disease (COPD), presence of stress ulcer, and treatment. Presence of COPD was defined and treated according to published guidelines (18, 19). Laboratory examinations included white blood cell count, red blood cell count, platelet count, serum calcium concentration, albumin, and blood glucose on admission. We calculated the neutrophil-lymphocyte ratio (NLR) (20), platelet-lymphocyte ratio (PLR) (20), prognostic nutritional index (PNI) (21), systemic immune-inflammation index (SII) (22) and SIRI according to the following equations: NLR = neutrophil count/lymphocyte count; PLR = platelet count/lymphocyte count; PNI = albumin +5 × total lymphocyte count; SII = (neutrophil count × platelet count)/lymphocyte count; and SIRI = (neutrophil count × monocyte count)/lymphocyte count.

Perihematomal edema volume was measured following the method of Gusdon et al. (23) using semiautomated planimetry with a three-dimensional slicer. Hematoma volume was calculated by the ABC/2 method (24). The mGS was calculated based on three factors, namely, location of intraventricular hemorrhage, hematoma volume in each ventricle, and ventricular dilatation, with a total score of 32 points (25). HE was defined as hematoma enlargement ≥6 mL or ≥ 33% within 24 h (26). The imaging evaluation was performed by two experienced neuroradiologists who were unaware of the other variables and outcomes. Data collection of laboratory results used the first-time examination at admission (within 24 h after admission). Surgical interventions mainly included hematoma evacuation with craniotomy and external ventricular drainage.

### Outcome assessment

All patients with HICH were treated stringently following the guidelines for the management of ICH (27). Patient outcome was evaluated by the Glasgow Outcome Scale (GOS) score at 3 months following HICH. A good outcome was defined as an GOS score of 4–5, and a poor outcome was defined as an GOS score of 1–3 (28).

### Statistical analysis

Statistical analyses were performed using SPSS 26.0 software (SPSS, INC; Chicago, United States), and nomogram establishment was undertaken by R 3.5.1 programming language.^1^ Categorical variables were presented as numbers with percentages and analyzed with an *x^2^* test or Fisher’s exact test, whereas continuous variables were expressed as mean ± standard deviation or median (interquartile range) and analyzed using Student’s *t*-test or Mann–Whitney *U* test. Logistic regression analyses were used to determine the influence of risk factors on outcomes in patients with HICH. Variables with *p* < 0.05 in univariate analysis were included in multivariate logistic regression. Since SII, NLR, and SIRI share common component factors, there may be bias when they were included together in multivariate logistic regression analysis. Therefore, only SIRI was included in multivariate logistic regression analysis in the study. The nomogram was constructed based on multivariable logistic regression results. The performance of the nomogram was assessed by discrimination and calibration. The discriminative ability of the nomogram was evaluated by the area under the receiver operating characteristic curve (AUC), which ranged from 0.5 (no discrimination) to 1 (perfect discrimination) (29). The calibration was evaluated using calibration curves and Hosmer-Lemeshow tests (30). Both discrimination and calibration were evaluated using bootstrapping methods with 1,000 resamples. Furthermore, decision curve analysis (DCA) was used to evaluate the net benefit of the model for patients (31). Two-sided *p*-values <0.05 was considered significant.

## Results

**Figure 1:**
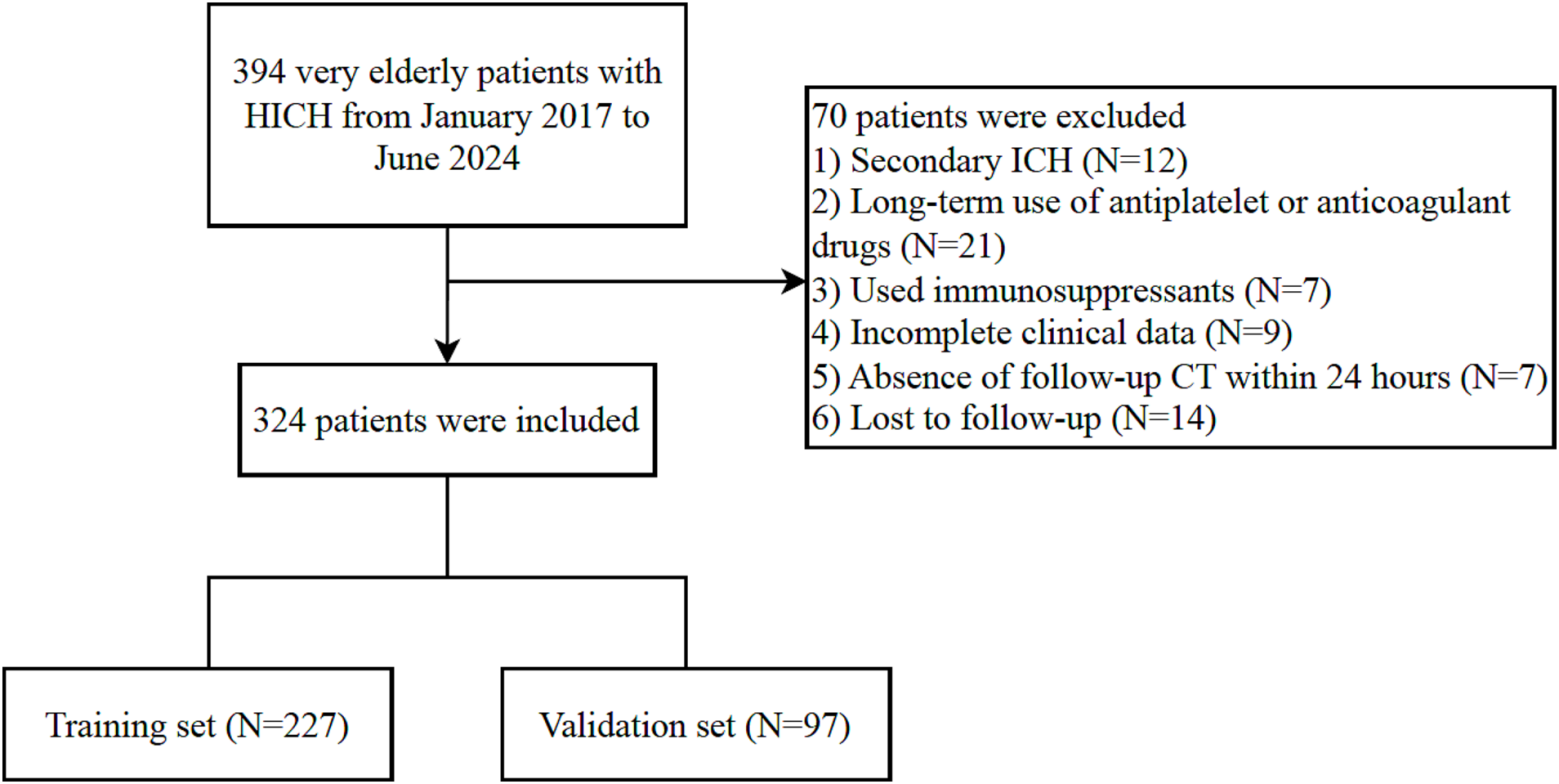
Flow chart for patient selection.

A total of 324 very elderly HICH patients were enrolled in this study, and they were randomly assigned to the training cohort (*n* = 227) and the validation cohort (*n* = 97) (Figure 1). Out of the 227 patients in the training group, 130 (57.3%) had poor outcomes and the mean age was 84 years. In contrast, 55 (56.7%) out of the 97 patients in the validation group had poor outcomes and the mean age was 84 years. The clinical characteristics of patients in the training and validation groups are summarized in Table 1.

**Table 1:** Characteristics and comparison between the training group and validation group.

Characteristic	Cohort (*n* = 324)		*p* value
	Training group (*n* = 227)	Validation group (*n* = 97)	
Gender, *n* (%)			0.790
Male	111 (48.9%)	49 (50.5%)	
Female	116 (51.1%)	48 (49.5%)	
Age (years)	84.7 ± 3.9	85.1 ± 4.1	0.411
Time from onset to CT (h)	2.8 (2.4, 5.0)	2.8 (2.2, 4.5)	0.713
Medical history, *n* (%)			
Diabetes mellitus	15 (6.6%)	5 (5.2%)	0.619
Coronary heart disease	25 (11.0%)	11 (11.3%)	0.932
GCS score at admission	13.0 (8.0, 14.0)	13.0 (9.0, 14.0)	0.930
Body temperature (°C)	36.5 (36.5, 36.7)	36.5 (36.4, 36.7)	0.536
Pulse (bpm)	76.0 (68.0, 85.0)	74.0 (66.0, 80.5)	0.071
Systolic blood pressure (mmHg)	182.0 (166.0, 198.0)	187.0 (164.5, 202.5)	0.332
Diastolic blood pressure (mmHg)	92.0 (83.0, 105.0)	95.0 (82.5, 103.0)	0.678
Hematoma location, *n* (%)			0.564
Supratentorial	206 (90.7%)	86 (88.7%)	
Infratentorial	21 (9.3%)	11 (11.3%)	
Hematoma volume (mL)	10.0 (5.0, 20.0)	12.0 (6.0, 20.0)	0.571
Perihematomal edema volume (mL)	12.0 (7.0, 22.0)	13.0 (7.0, 22.0)	0.643
mGS	0.0 (0.0, 6.0)	0.0 (0.0, 6.0)	0.787
Hematoma expansion, *n* (%)	97 (42.7%)	39 (40.2%)	0.673
Treatment, *n* (%)			0.653
Surgical intervention	22 (9.7%)	11 (11.3%)	
Conservative treatment	205 (90.3%)	86 (88.7%)	
COPD, *n* (%)	111 (48.9%)	50 (51.5%)	0.662
Presence of stress ulcer, *n* (%)	54 (23.8%)	27 (27.8%)	0.441
Laboratory examination			
White blood cell count (10^9^/L)	9.1 (6.5, 12.2)	8.2 (6.6, 11.1)	0.191
Red blood cell count (10^12^/L)	4.1 (3.8, 4.5)	4.1 (3.8, 4.3)	0.250
Platelet count (10^9^/L)	202.0 (158.0, 233.0)	202.0 (152.5, 233.0)	0.520
Calcium (mmol/L)	2.2 (2.1, 2.3)	2.2 (2.1, 2.3)	0.479
Albumin (g/L)	40.1 (37.2, 42.1)	41.0 (38.1, 42.3)	0.152
Glucose (mmol/L)	7.0 (5.9, 8.9)	6.8 (5.6, 9.1)	0.758
NLR	6.6 (3.1, 12.7)	5.4 (2.3, 10.9)	0.033
PLR	177.6 (118.6, 270.9)	159.2 (89.1, 254.9)	0.062
PNI	45.7 (41.8, 49.5)	46.1 (43.8, 51.7)	0.057
SII	1164.1 (608.0, 2808.0)	879.0 (453.9, 2162.0)	0.017
SIRI	3.1 (1.3, 5.2)	2.1 (0.9, 4.8)	0.035
Functional outcome, *n* (%)			0.925
Poor	130 (57.3%)	55 (56.7%)	
Good	97 (42.7%)	42 (43.3%)	

CT, computed tomography; GCS, Glasgow Coma Scale; mGS, modified Graeb Score; COPD, chronic obstructive pulmonary disease; NLR, neutrophil-lymphocyte ratio; PLR, platelet-lymphocyte ratio; PNI, prognostic nutritional index; SII, systemic immune-inflammation index; SIRI, systemic inflammation response index.

**Figure 2:**
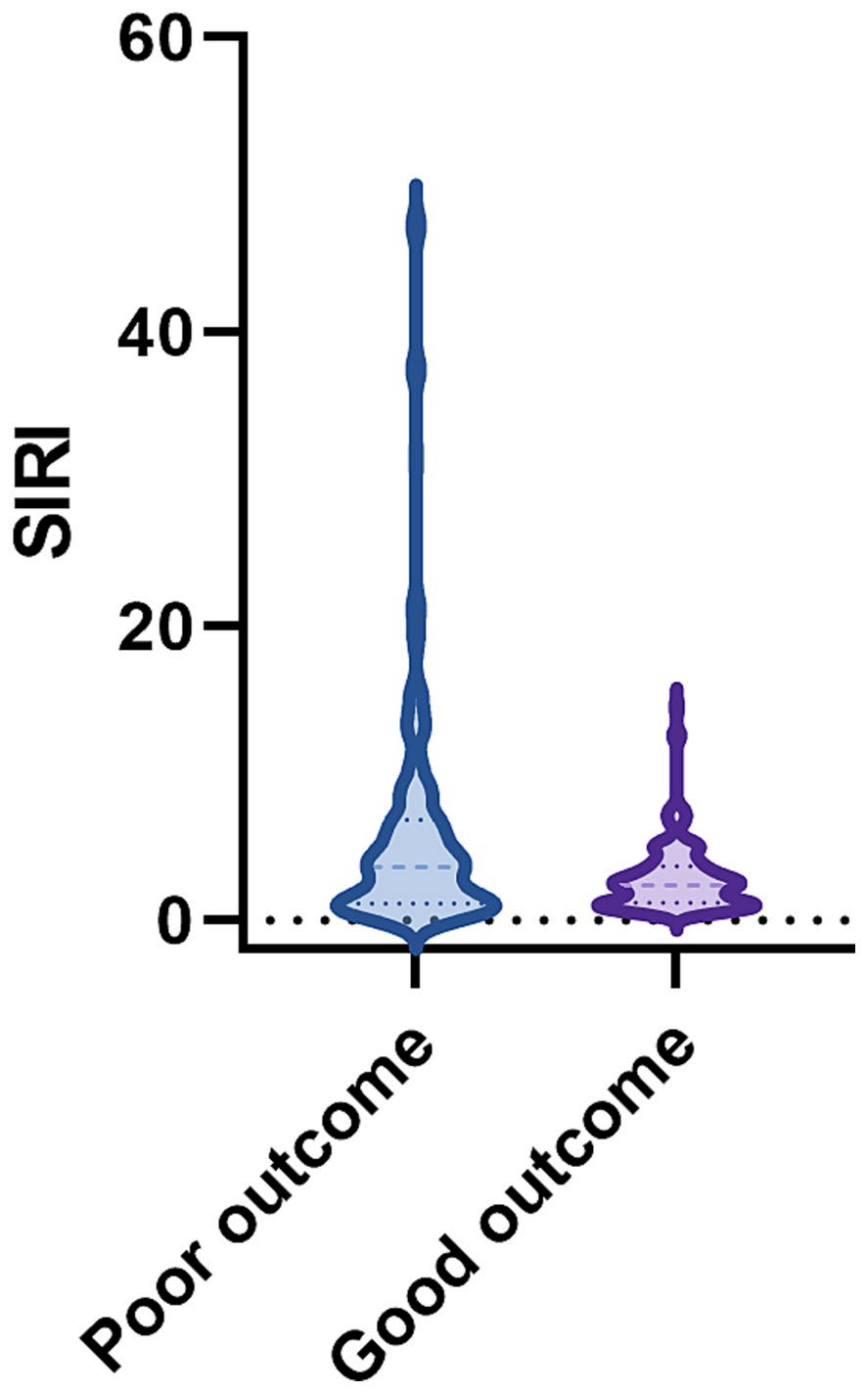
Boxplots of SIRI showing the distribution in the good outcome group (*n* = 139) and poor outcome group (*n* = 185). The SIRI of the poor outcome group was higher than that of the good outcome group (<0.001).

**Figure 3:**
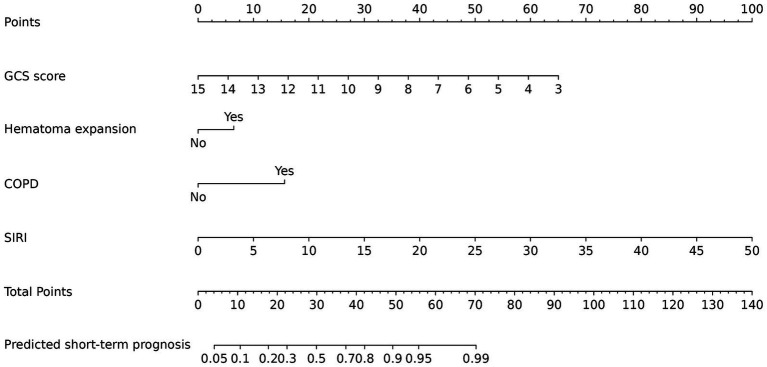
Predictive nomogram model for assessing the short-term prognosis in very elderly patients with hypertensive intracerebral hemorrhage.

In the training group, the data of 227 patients were used to establish the nomogram. Of these patients, 130 (57.3%) had poor outcomes. Between good outcomes and poor outcomes, there were significant differences in time from onset to CT (*p* < 0.001), history of coronary heart disease (*p* < 0.001), GCS score (*p* < 0.001), systolic blood pressure (*p* < 0.001), baseline HICH volume (*p* < 0.001), perihematomal edema volume (*p* < 0.001), mGS (*p* < 0.001), hematoma expansion (*p* < 0.001), COPD (*p* < 0.001), presence of stress ulcer (*p* < 0.001), white blood cell count (*p* < 0.001), blood glucose (*p* = 0.002), NLR (*p* = 0.020), SII (*p* = 0.016), and SIRI (*p* < 0.001) (Table 2). Patients in the poor outcome group showed significantly higher SIRI values, as shown in Figure 2. A multivariate logistic regression analysis revealed that GCS score (odds ratio [OR], 1.968; 95% confidence interval [CI], 1.489–2.602; *p* < 0.001), hematoma expansion (OR, 0.216; 95% CI, 0.047–0.992; *p* = 0.049), COPD (OR, 0.193; 95% CI, 0.055–0.677; *p* = 0.010), and SIRI (OR, 0.709; 95% CI, 0.558–0.900; *p* = 0.005) were independent risk factors for poor outcome of HICH (Table 3). Based on the four independent predictors of poor outcome identified by multivariate logistic regression modeling, we constructed the nomogram for poor outcome in very elderly HICH patients (Figure 3). The predicted probability of poor outcome can be derived after adding up the scores corresponding to each variable and locating them on the total point scale.

**Table 2:** Characteristics and comparison between the good outcome group and poor outcome group in training cohort.

Characteristics	Good outcome	Poor outcome	*p* value
Number of patients	97	130	
Gender, *n* (%)			0.220
Male	52 (53.6%)	59 (45.4%)	
Female	45 (46.4%)	71 (54.6%)	
Age (years)	84.9 ± 4.2	84.5 ± 3.7	0.611
Time from onset to CT (h)	3.5 (2.5, 6.2)	2.6 (2.0, 4.4)	<0.001^*^
Medical history, *n* (%)			
Diabetes mellitus	5 (5.2%)	10 (7.7%)	0.446
Coronary heart disease	19 (19.6%)	6 (4.6%)	<0.001^*^
GCS score at admission	14.0 (13.0, 15.0)	9.0 (5.0, 13.0)	<0.001^*^
Body temperature (°C)	36.5 (36.5, 36.7)	36.5 (36.5, 36.7)	0.603
Pulse (bpm)	76.0 (67.0, 85.0)	78.0 (70.0, 85.0)	0.345
Systolic blood pressure (mmHg)	170.0 (162.0, 190.0)	188.0 (179.0, 201.0)	<0.001^*^
Diastolic blood pressure (mmHg)	89.0 (83.0, 97.0)	94.0 (84.0, 105.0)	0.053
Hematoma location, *n* (%)			0.161
Supratentorial	85 (87.6%)	121 (93.1%)	
Infratentorial	12 (12.4%)	9 (6.9%)	
Hematoma volume (mL)	5.0 (4.5, 9.0)	20.0 (10.0, 40.0)	<0.001^*^
Perihematomal edema volume (mL)	7.0 (6.0, 10.5)	21.0 (12.0, 42.0)	<0.001^*^
mGS	0.0 (0.0, 0.0)	4.0 (0.0, 13.0)	<0.001^*^
Hematoma expansion, *n* (%)	17 (17.5%)	80 (61.5%)	<0.001^*^
Treatment, *n* (%)			0.239
Surgical intervention	12 (12.4%)	10 (7.7%)	
Conservative treatment	85 (87.6%)	120 (92.3%)	
COPD, *n* (%)	15 (15.5%)	96 (73.8%)	<0.001^*^
Presence of stress ulcer, *n* (%)	5 (5.2%)	49 (37.7%)	<0.001^*^
Laboratory examination			
White blood cell count (10^9^/L)	7.8 (6.3, 10.9)	9.9 (7.0, 14.2)	<0.001^*^
Red blood cell count (10^12^/L)	4.2 (3.9, 4.4)	4.1 (3.7, 4.7)	0.678
Platelet count (10^9^/L)	188.0 (153.0, 235.0)	205.0 (160.0, 231.8)	0.349
Calcium (mmol/L)	2.2 (2.1, 2.3)	2.2 (2.1, 2.3)	0.749
Albumin (g/L)	39.0 (36.0, 41.8)	40.5 (38.0, 42.3)	0.054
Glucose (mmol/L)	6.2 (5.4, 8.4)	7.6 (6.4, 8.9)	0.002^*^
NLR	5.6 (2.8, 10.6)	9.3 (3.4, 13.3)	0.020^*^
PLR	158.6 (120.8, 254.7)	200.0 (118.0, 290.7)	0.303
PNI	45.0 (41.5, 49.8)	46.0 (42.7, 49.3)	0.182
SII	1005.8 (550.6, 1848.2)	1615.9 (749.3, 3113.7)	0.016^*^
SIRI	2.5 (1.2, 3.8)	3.8 (1.4, 7.3)	<0.001^*^

CT, computed tomography; GCS, Glasgow Coma Scale; mGS, modified Graeb Score; COPD, chronic obstructive pulmonary disease; NLR, neutrophil-lymphocyte ratio; PLR, platelet-lymphocyte ratio; PNI, prognostic nutritional index; SII, systemic immune-inflammation index; SIRI, systemic inflammation response index. * *p* < 0.05 was considered significant.

**Table 3:** Multivariate logistic regression analysis of predictors for poor outcome.

Variable	B value	OR	95% CI	*p* value
Time from onset to CT	0.053	1.055	0.894–1.244	0.526
History of coronary heart disease	−1.291	0.275	0.050–1.509	0.137
GCS score	0.677	1.968	1.489–2.602	<0.001^*^
Systolic blood pressure	−0.009	0.991	0.973–1.010	0.346
Hematoma volume	0.785	2.193	0.814–5.906	0.120
Perihematomal edema volume	−0.843	0.430	0.160–1.160	0.096
mGS	0.116	1.123	0.975–1.293	0.109
Hematoma expansion	−1.530	0.216	0.047–0.992	0.049^*^
COPD	−1.645	0.193	0.055–0.677	0.010^*^
Presence of stress ulcer	−0.658	0.518	0.113–2.367	0.396
White blood cell count	0.185	1.203	0.983–1.473	0.073
Glucose	0.081	1.084	0.867–1.355	0.478
SIRI	−0.344	0.709	0.558–0.900	0.005^*^

OR, odds ratio; CI, confidence interval; CT, computed tomography; GCS, Glasgow Coma Scale; mGS, modified Graeb Score; COPD, chronic obstructive pulmonary disease; SIRI, systemic inflammation response index. * *p* < 0.05 was considered significant.

**Figure 4:**
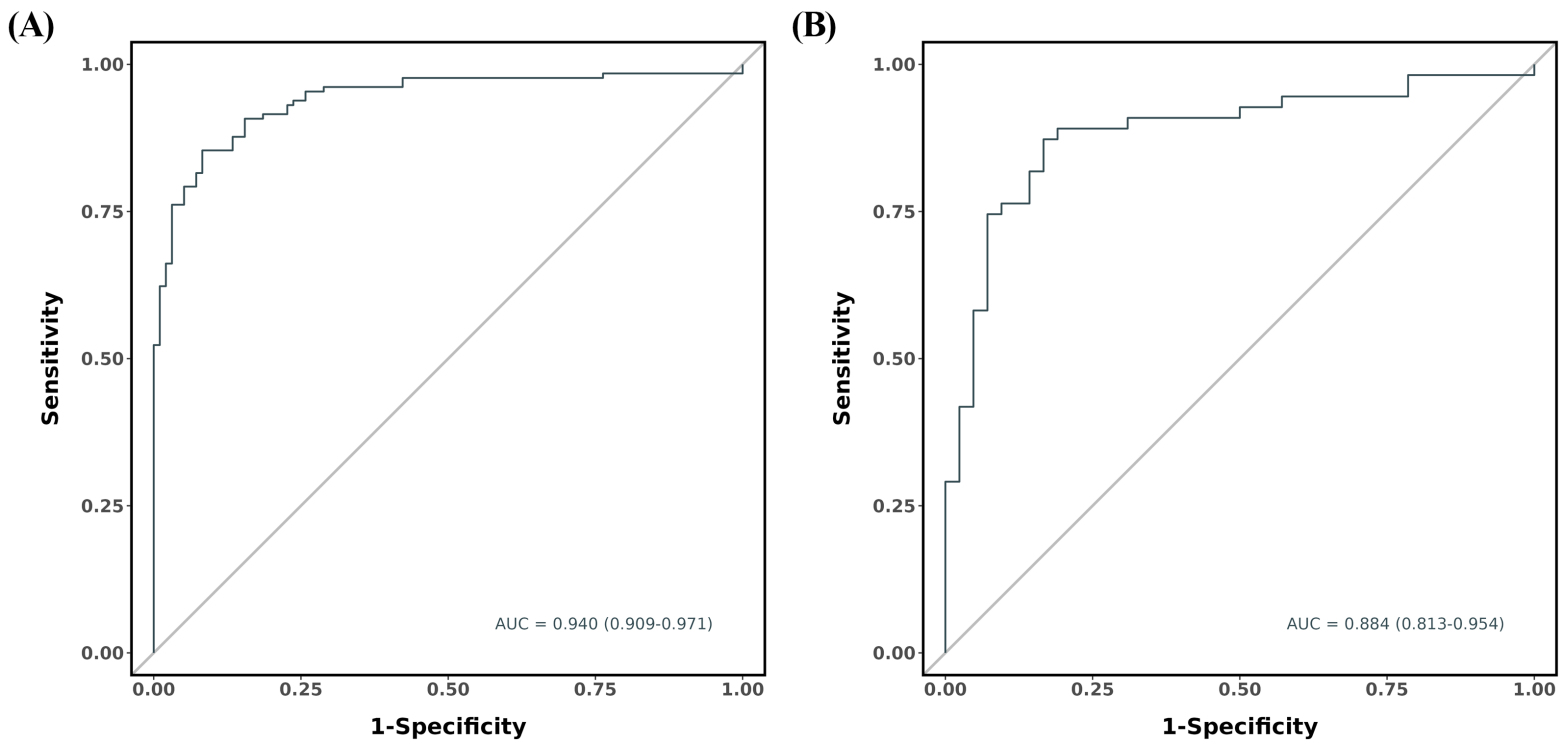
Receiver operating characteristic curve of predicting the short-term prognosis in very elderly patients with hypertensive intracerebral hemorrhage by the nomogram model. Training group **(A)**, validation group **(B)**.

**Figure 5:**
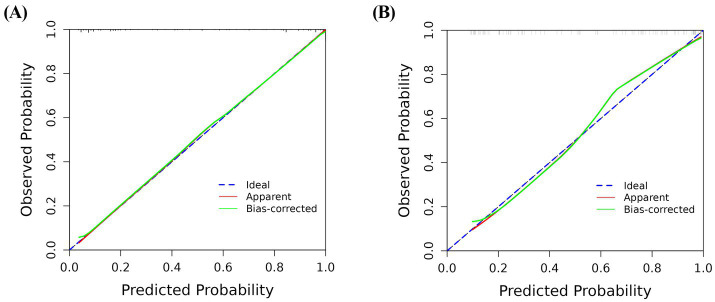
Calibration curve for predicting the short-term prognosis in very elderly patients with hypertensive intracerebral hemorrhage by the nomogram model. Training group **(A)**, validation group **(B)**.

**Figure 6:**
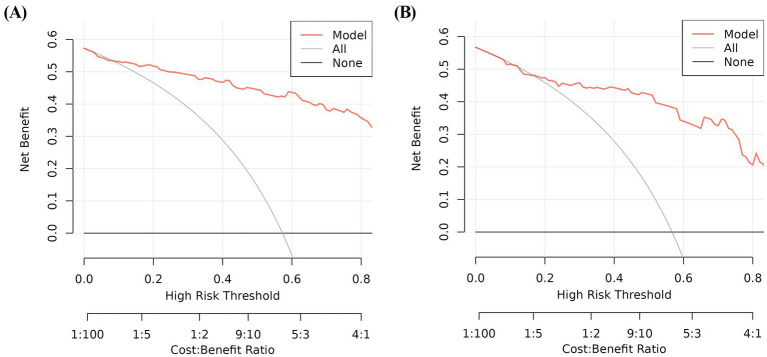
Decision curve analysis for predicting the short-term prognosis in very elderly patients with hypertensive intracerebral hemorrhage by the nomogram model. Training group **(A)**, validation group **(B)**.

In the training and validation cohorts, the AUC under the receiver operating characteristic curve was 0.940 (95% CI: 0.909, 0.971), and 0.884 (95% CI: 0.813, 0.954), respectively, which indicated that the nomogram had a good discriminative ability (Figure 4). Calibration curves confirmed that the probabilistic forecasting of poor outcome aligned with the observed integration value for both the training and validation cohorts (Figure 5). The Hosmer-Lemeshow test showed no statistical significance in the training (*p* = 0.799) and validation (*p* = 0.644) cohorts. A DCA revealed that the nomogram for predicting the short-term prognosis provided greater net benefit than the treat-all-patients and the treat-none-patients in the training and validation cohorts, which suggested that the nomogram had high clinical utility in practical applications (Figure 6).

## Discussion

This study demonstrated that GCS score, hematoma expansion, COPD, and SIRI were significant predictors of poor outcome in very elderly HICH patients. Recently, although some studies have established different prognostic prediction models on patients with HICH, there are relatively few studies that focus on very elderly patients with HICH. In view of this, we included very elderly patients with HICH from two centers and constructed a nomogram based on SIRI and clinical risk factors for evaluating short-term prognosis. The nomogram showed good performance in training and validation cohorts and was an easy-to-use personalized decision-making tool.

Neuroinflammation has been linked to neurological diseases, especially cerebrovascular disease (32). Previous studies have demonstrated that inflammatory response plays a crucial role in the pathologic mechanism of ICH and is associated with poor functional outcome (33). There is increasing evidence that easily available serum biomarkers of inflammation can serve as reliable predictors of outcome in patients with ICH and can improve the outcome prediction when added to validated prognostic scales (34). The SIRI is a novel biomarker of inflammatory process. A few studies have found that in patients with ischemic stroke and traumatic spinal cord injury, the mortality rate of patients with high SIRI is higher than that of patients with low SIRI (35, 36). Wang et al. (37) have investigated the relationship between SIRI and clinical outcome of ICH, and found that the SIRI was the best predictor for poor outcomes at discharge in ICH patients. In our study, both univariate and multivariate logistic analyses revealed that SIRI was positively correlated with an unfavorable outcome following HICH. A high level of inflammatory factors such as NLR and PLR are associated with poor outcome of ICH (38, 39). The prevalence of ICH is high in the elderly, chronic and complex comorbidities among whom are somewhat common. SIRI is an important inflammatory indicator, which can be used to evaluate the severity of the comorbidities (40). An elevation of the SIRI is associated with the short-term prognosis of elderly HICH.

Consistent with the previous reports, we found that the admission GCS score was associated with poor clinical outcome. Øie et al. (41) reported that a GCS score of <9 on admission was a risk factor for poor prognosis at 3 months in patients with ICH. In addition, the hematoma expansion could be a predictor of poor outcome, which was similar to the results of Morotti and colleagues (42). It was found that HE was associated with a greater risk of poor outcome in ICH. HE is a common early and severe complication of ICH (43, 44). Several studies indicate that HE occurs in approximately one-third of patients with ICH and is associated with in-hospital mortality and poor outcome (45, 46). Believed to occur hours after symptom onset and initial hemorrhage, HE occurs with an increase in intracranial pressure, resulting in mass effect, midline shift, and herniation (47). In addition, it is also one of the main causes of secondary brain injury, as iron and heme from lysed erythrocytes create a highly oxidative and cytotoxic environment, damaging brain tissue (48, 49). These all lead to significant neurological deficits and worse functional outcomes following ICH. It is well recognized that ICH outcomes differ significantly depending on their location (50, 51). Traditionally, ICH prognostication based on location is separated crudely into infratentorial and supratentorial (52). However, recent studies have demonstrated that the prognostication of ICH outcomes should be classified into more specific anatomic sites (53, 54). Although studies have produced inconsistent results, for supratentorial ICH, the prognosis is generally better for lobar ICH (51, 55, 56) but worse for thalamic (57, 58). Importantly, since ICH volume is also another crucial modifier of ICH outcomes, it is becoming more evident that a location-specific ICH volume would better predict ICH outcomes (59). A small strategic bleed affecting the thalamus may have devastating neurological deficits, while a similar-sized ICH at the frontal lobe could have a more favorable neurological outcome (60). In addition, the risk of HE is also associated with the hemorrhage location. Thalamic ICH had a smaller volume at baseline CT and a low risk of HE, but lobar ICH was likely to develop HE (61, 62). Therefore, as the interaction between ICH location and volume significantly impacts neurological outcomes, using location-specific hematoma volumes for patient selection could better guide the selection of patients who may best benefit from treatment.

The findings of current study revealed the role of COPD in short-term prognosis. COPD is a common disease in the elderly, which has been reported to be detrimental for patients with ICH (63). The mechanism of COPD in poor outcomes may be because of ventilator-associated pneumonia. COPD has an adverse impact on respiratory muscle function, and it may be amplified during neurocritical illness (64). Ozyuvaci et al. (65) reported that patients with COPD exhibited an increased partial pressure of carbon dioxide (PaCO_2_) that reached the highest level after extubation and did not return to baseline levels. Besides, COPD is an inflammatory disease with complex pathobiology where various underlying mechanisms are implicated (66, 67). Increased numbers of inflammatory cells such as macrophages, neutrophils, and lymphocytes have been reported in COPD patients which play critical roles in the development and pathogenesis of COPD inflammation, directly contributing to airway remodeling and a decrease in lung function (68). Makris et al. (69) has been reported COPD is associated with longer intensive care unit stay and mortality. The present study also revealed that COPD was an independent predictor for poor prognosis in elderly HICH patients. Therefore, very elderly patients with HICH who present with COPD should be monitored closely for any fluctuations in PaCO_2_ during hospitalization, and appropriate clinical interventions should be employed to enhance patient prognosis.

Early identification and treatment is important for preventing the development of poor prognosis. This is best accomplished by identifying high-risk patients. Consequently, these patients need to be under observation, followed up, and should undergo early intervention. The nomogram is one of the methods to present the predicted model as the graphic scoring. After performing the multivariate regression analysis, we included GCS score, hematoma expansion, COPD, and SIRI, a total of four factors, as nomogram score points. By combining SIRI with clinical features, a novel nomogram was developed to promote further clinical application and accurately predict the prognosis of HICH. Compared with a single index or risk factor, this nomogram has further improved the performance of predicting the prognosis of HICH and achieved higher accuracy. The AUCs of the training and validation cohorts were 0.940 and 0.884, respectively. Furthermore, the calibration curve and DCA curve showed that the nomogram had good consistency and potential clinical applicability, and the maximum benefit was obtained under all thresholds. The doctor can add the scores of each prediction variable and get the total score according to the individual differences of patients, so as to better help clinical decision-making and enable clinicians to develop personalized treatment plans for elderly HICH patients.

This study has several limitations. First, this was a retrospective analysis and may therefore have a certain degree of selection bias. Second, the sample size of this study is not very large. Third, blood parameters were recorded only once on admission and were not monitored dynamically. Fourth, our model only focuses on the short-term prognosis of elderly patients with HICH, which, although important for clinical practice, does not assess long-term prognosis. Last, the prediction model was only subjected to internal validation, lacking external validation, it is necessary to conduct large sample and multicenter studies to prove the feasibility of the nomogram and increase the possibility of extensive popularization of the model.

## Conclusion

The results of this study indicate that SIRI is an independent risk factor for poor outcome. The nomogram including GCS score, hematoma expansion, COPD, and SIRI was reliable for predicting the short-term prognosis of very elderly patients with HICH.

## Data Availability

The original contributions presented in the study are included in the article/supplementary material, further inquiries can be directed to the corresponding authors.

## Footnotes

^1^ https://www.r-project.org
